# Draft Genome Sequence of *Lactobacillus delbrueckii* Strain #22 Isolated from a Patient with Short Bowel Syndrome and Previous d-Lactic Acidosis and Encephalopathy

**DOI:** 10.1128/genomeA.00747-16

**Published:** 2016-07-28

**Authors:** Eugen Domann, Florence Fischer, Fabian Glowatzki, Moritz Fritzenwanker, Torsten Hain, Silke Zechel-Gran, Susanne Giffhorn-Katz, Bernd A. Neubauer

**Affiliations:** a Institute for Medical Microbiology, German Centre for Infection Research (DZIF Partner Site Giessen-Marburg-Langen), Justus-Liebig-University Giessen, Giessen, Germany; b Institute for Medical Microbiology and Hygiene, German Centre for Infection Research (DZIF Partner Site Giessen-Marburg-Langen), Philipps University Marburg, Marburg, Germany; c Olgahospital Stuttgart, Stuttgart, Germany; d Institute of Laboratory Medicine, Pathobiochemistry and Molecular Diagnostics, Justus-Liebig-University Giessen, Giessen, Germany; e Department of Neuropediatrics, Justus-Liebig-University Giessen, Giessen, Germany

## Abstract

d-Lactic acidosis with associated encephalopathy caused by overgrowth of intestinal lactic acid bacteria is a rarely diagnosed neurological complication of patients with short bowel syndrome. Here, we report the draft genome sequence of *Lactobacillus delbrueckii* strain #22 isolated from a patient with short bowel syndrome and previous d-lactic acidosis/encephalopathy.

## GENOME ANNOUNCEMENT

In patients with short bowel syndrome (SBS), colonic fermentation of malabsorbed or unabsorbed carbohydrates produces high amounts of organic acids, such as d-/l-lactic acid and short-chain fatty acids, resulting in lowered luminal pH. This may promote the growth of a high concentration of lactic acid bacteria (LAB), including d-lactic acid-producing bacteria of the genus *Lactobacillus*. Additionally, taking antibiotics or probiotics or ingesting fermented food (e.g., yogurt, sauerkraut, and pickled vegetables) can increase the concentration of these bacteria in the colon. A high production of d-lactic acid may then occur following the ingestion of large amounts of carbohydrates, particularly readily fermentable simple sugars. d-lactic acidosis occurs because d-lactic acid can hardly be excreted or metabolized by humans. Typical clinical symptoms in patients with SBS and d-lactic acidosis comprise slurred speech, ataxia, altered mental status, somnolence, and even coma (1–5). We have isolated a *Lactobacillus delbrueckii* strain, designated *L. delbrueckii* #22, from a patient with SBS a few days after an acute episode of d-lactic acidosis and encephalopathy. The *in vitro* analysis of the culture supernatant of strain #22 revealed the production of ~98% d-lactic acid and ~2% l-lactic acid, as determined by the Enzytec d-/l-lactic acid enzymatic assay distributed by R-Biopharm AG, Darmstadt, Germany (6). Written informed consent was obtained from the patient for the study. The study was approved by the ethics board of the Justus-Liebig-University of Giessen, Germany (AZ: 192/09).

*Lactobacillus* spp. are important members of the LAB group and comprise a large and heterogeneous genus of microaerobic, catalase-negative, non-spore-forming, Gram-positive rods, which produce lactic acid as their single or major metabolic end product from glucose fermentation. Lactobacilli are Janus-faced bacteria that are generally regarded as safe (GRAS) by the U.S. Food and Drug Administration (FDA) and are widely used for the production of fermented food. Nevertheless, they are able to cause even severe infections, especially in immunocompromised individuals (7–9).

DNA sequencing libraries were prepared using the Nextera XT kit (Illumina, San Diego, CA), according to the manufacturer’s instructions. Individually tagged libraries were sequenced as a part of a flow cell on the Illumina MiSeq platform with v2 chemistry (2 × 250 bp; Illumina). A total of 6,588,530 paired sequences with an average length of 134 bp were produced and assembled to 10 contigs, with a total length of 2,188,982 bp, using CLC Genomics Workbench version 7.0.4. Contigs were ordered to reference strain *L. delbrueckii* subsp. *bulgaricus* ND02 (10) using MAUVE (11) and annotated by GenDB (12).

The genome analysis revealed three copies of d-lactate dehydrogenases and one L-lactate dehydrogenase. This constellation is a potential explanation for the relative abundance of d-/l-lactic acid concentration in the bacterial supernatant.

### Nucleotide sequence accession number.

This whole-genome shotgun project has been deposited in the European Nucleotide Archive under the accession no. FLLT01000000. The version described in this paper is the first version.
